# Interleukin-25 Mediated Induction of Angiogenin-4 Is Interleukin-13 Dependent

**DOI:** 10.1371/journal.pone.0153572

**Published:** 2016-04-18

**Authors:** Zannatun Noor, Stacey L. Burgess, Koji Watanabe, William A. Petri

**Affiliations:** 1 Division of Infectious Diseases and International Health, Department of Medicine, University of Virginia Health System, Charlottesville, Virginia, United States of America; 2 AIDS Clinical Center, National Center for Global Health and Medicine, Tokyo, Japan; University of Wisconsin Medical School, UNITED STATES

## Abstract

The intestinal surface is directly exposed to both commensal microorganisms as well as pathogens with a single layer of epithelium separating luminal microorganisms from internal tissues. Antimicrobial peptides play a crucial role in allowing epithelial cells to contain in the lumen beneficial and pathogenic microorganisms. The commensal dependent, epithelial produced, Th2 cytokine IL-25 can induce IL-13 and potentially the antimicrobial peptide angiogenin-4. Here we show that IL-13 downstream of IL-25 is required to induce angiogenin-4. IL-25 mediated induction of angiogenin-4 is furthermore not dependent on IL-22 or IL-17.

## Introduction

The intestinal epithelial layer provides a physical barrier which separates commensal and pathogenic microorganisms from submucosal tissue. It maintains homeostatic relationships between host and commensal microorganism by means of limiting antigenic and pathogenic exposure. Epithelial cells play an important role in this intestinal homeostasis by secreting cytokines, mucus and antimicrobial peptides. Interleukin-25 is a Th2 associated cytokine often produced alongside IL-4, IL-5, IL-13 and IL-9 [1,2]. IL-25 is secreted from gut epithelial cells following stimulation by commensal bacteria, and IL-25 suppresses the IL-23-IL-17 axis to control gut inflammation [3]. However, the role and mechanism of IL-25 in induction of antimicrobial peptides has not been clearly defined. Antimicrobial peptides play an important role in control of the commensal bacteria in the gut, and provide defense against pathogens. IL-22, which is induced by IL-23, is well known to trigger the secretion of antimicrobial peptides from Paneth cells [4]. However, it is unlikely that IL-25 acts via IL-23, as IL-23 secretion is suppressed by IL-25 [3]. Studies have shown that the Th2 cytokine IL-13 induces Paneth and goblet cells to produce an antimicrobial peptide, angiogenin-4 [5]. Here we show that IL-25 is a potent inducer of the antimicrobial peptide angiogenin-4, and acts in an IL-13 dependent manner. This work therefore helps better explain the role of IL-25 in protecting the gut barrier via antimicrobial peptide production.

Angiogenin-4 induces blood vessel formation and is a member of the ribonuclease family of proteins. Its activity as an antimicrobial peptide is more recently known [6]. During *Salmonella* challenge, IL-23 induces IL-22 production which triggers Paneth cells to produce angiogenin-4 [7]. During *Trichuris muris* infection, angiogenin-4 expression is correlated with worm expulsion [8]. During *Trichinella spiralis* infection, worm expulsion accompanied IL-25 mediated host protection and IL-25 induces angiogenin-4 expression [9]. Angiogenin-4 is well known as a Paneth cell-derived antimicrobial peptide, however it is also known that it is produced by goblet cells during *Trichuris muris* infection under control of IL-13 [5]. Previous studies have shown that it is regulated by IL-9 and requires IL-13, not IL-4 [10]. However, there is not clear evidence that explains how IL-25 induces angiogenin-4 production. Here, we show that IL-25 induces angiogenin-4 production in an IL-13 dependent manner, rather than via IL-22 or IL-17.

## Materials and Methods

### Mice

Six week old male CBA/J mice (Jackson Laboratories) were housed in a specific pathogen–free facility in micro isolator cages and provided autoclaved food (Lab diet 5010) and water ad libitum. The University of Virginia Institutional Animal Care and Use Committee approved all procedures. The physical condition of animals were monitored once a day and no animals became ill or died prior to the experimental endpoint. We do have a protocol for the use of humane endpoints and animals were euthanized using carbon dioxide.

### Recombinant IL-25 or rIL-13 treatment and cecal tissue collection

Mice were injected intraperitoneally with 0.5 micrograms of recombinant IL-25 (RnD system) or PBS in a 100 microliter volume each day for 4–10 days. Recombinant IL-13 was injected each day for a total of four doses. Mice were harvested to collect cecal tissue.

### Quantitative real-time RT-PCR

Total RNA was isolated from cecal tissue using RNeasy Mini Kit (Qiagen, Hilden, Germany) and cDNA was generated using the tetro cDNA synthesis kit (Bioline USA Inc. USA). Mouse angiogenin-4 and IL-13 gene expression was measured by real-time PCR using Sybr green with normalization to expression of the mouse house keeping genes βActin and GAPDH. IL-13 primers were purchased from Qiagen (Hilden, Germany). Angiogenin-4 primer sequences were: Angiogenin-4 forward: 5’- TTGGCTTGGCATCATAGT -3’, Angiogenin-4 reverse: 5’- CCAGCTTTGGAATCACTG -3’, Data were normalized with house keeping gene βActin; βActin Forward: 5’- AGCCATGTACGTAGCCATCC-3’, βActin Reverse: 5’-CTCTCAGCTGTGGTGGTGAA -3’, and GAPDH; GAPDH Forward: 5’-TGCACCACCAACTGCTTAGC -3’, GAPDH Reverse: 5’-GGCATGGACTGTGGTCATGAG -3’. Primers were purchased from Integrated DNA Technologies Coralville, Iowa, USA.

### Immunohistochemistry and scoring

Cecum tissue were fixed with Bouin’s solution (Sigma-ALDRICH, St. Louis, MO) and paraffin embedded sections of cecum were cut into four micron histologic sections, placed on charged glass slides (Superfrost Plus, Fisher Scientific, Pittsburgh, PA). Then slides were deparaffinized and antigen retrieval was performed in PT Link instrument (Dako, Glostrup, Denmark) at 97°C for 20 minutes in low pH antigen retrieval solution. Immunohistochemistry was done on a robotic platform (Autostainer, Dako). Endogenous peroxidases were blocked using Peroxidase and Alkaline Phosphatase Blocking Reagent (Dako). Polyclonal rabbit antibody to Angiotensin 4 (obtained from Dr. Lora Hooper, Univ. Texas Southwestern Medical Center, Dallas, TX) was diluted 1:2,000, and applied at ambient temperature for 60 minutes. Antibody binding was visualized by incubation with Envision^™^ Rabbit Link (Dako) and then incubated with 3,3’-diaminobenzidine tetrahydrochloride (DAB+). All the slides were counterstained with hematoxylin subsequently; they were dehydrated, cleared and mounted for the assessment. Immunohistochemistry scoring was done blindly. The scoring was as 1 to 5 from low to high Angiogenin-4 protein staining.

### Antibody Neutralization

6 week old male CBA/J mice were treated with 0.5 micrograms of recombinant IL-25 each day for a total of 7 doses and control mice received PBS. Recombinant IL-25 treated mice received 200μg anti IL-17 (Amgen) or 200μg anti IL-22 antibody (Genentech) or 200μg anti-IL-13 antibody (Genentech) or isotype control on day 3, on day 5 and on day 7. Mice were sacrificed one day after the last injection.

### Statistical analysis

Student's *t*-test or Mann-Whitney non-parametric *t*-test was used for comparisons between two groups. *P* values of less than 0.05 were considered significant. Statistical analysis was presented using GraphPad Prism, GraphPad Software, San Diego California, USA. All experiments are representative of at least two independent replicates.

## Results

### rIL-25 administration induces angiogenin-4 expression in a dose dependent manner

**Fig 1:**
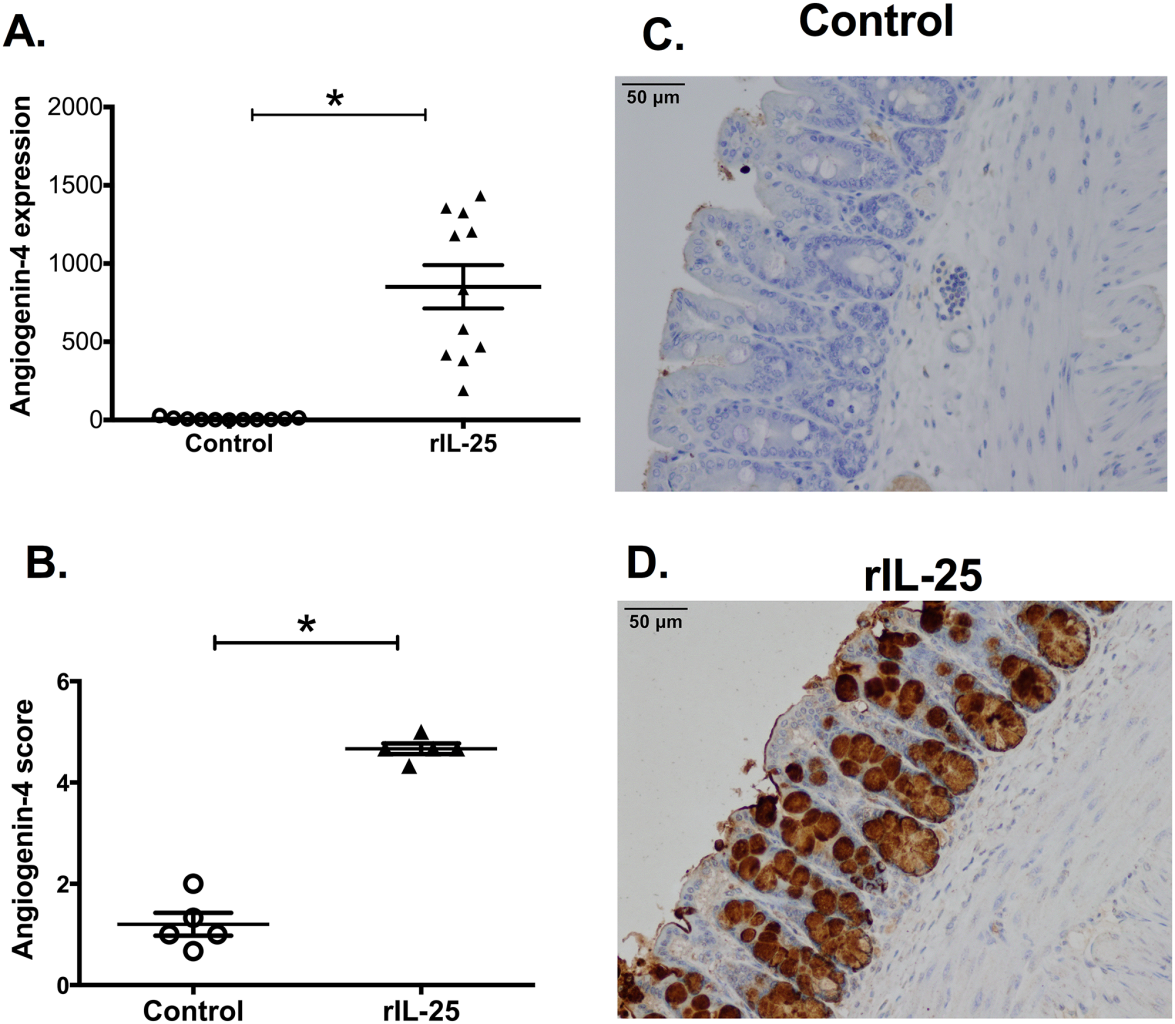
rIL-25 administration induces angiogenin-4 expression. CBA/J mice were treated with 0.5 micrograms of recombinant IL-25 (triangle, n = 11) each day for a total of 10 doses over 10 days. Control mice received PBS (open circle, n = 11). Angiogenin-4 relative expression was measured from mouse cecal tissue and normalized with house keeping gene GAPDH and β actin (A). Histological scoring (1 to 5; low to high) for Angiogenin-4 in cecum from mice treated with PBS or rIL-25 (B). Representative IHC staining for angiogenin-4 in samples of cecum tissue from PBS treated (C) or rIL-25 treated mice (D).

**Fig 2:**
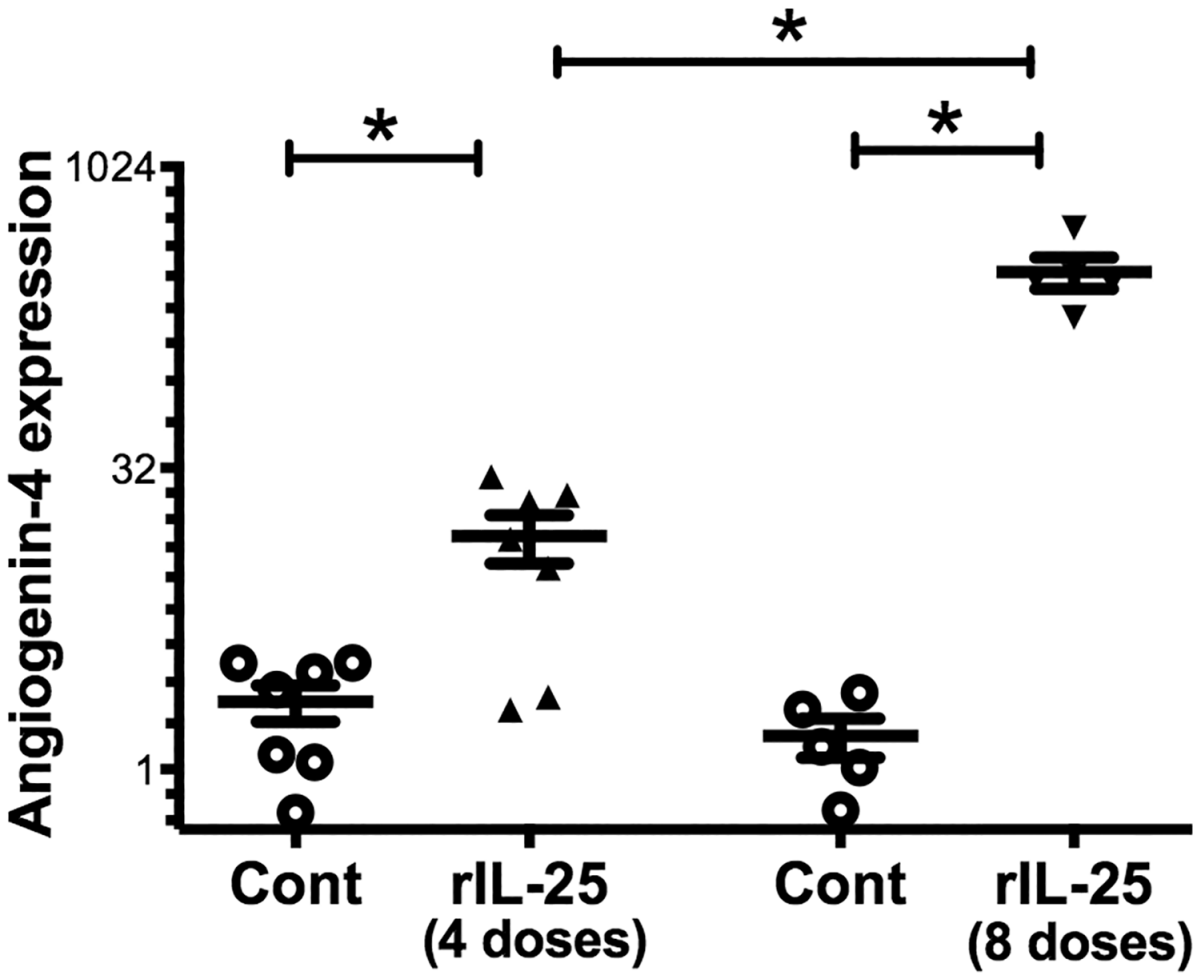
rIL-25 administration increases angiogenin-4 expression in a dose dependent manner. CBA/J mice were treated with 0.5 micrograms of rIL-25 each day for a total 4 doses (triangle, n = 7) or 8 doses (inverse triangle, n = 5) and control mice received 4 or 8 doses of PBS (open circle, n = 7 for 4 doses and n = 5 for 8 doses). Angiogenin-4 relative expression was measured from mouse cecal tissue and normalized with house keeping gene GAPDH and β actin.

In order to test if IL-25 induces angiogenin-4 production, we treated CBA/J mice with recombinant IL-25 (rIL-25), and then we measured in mouse ceca the mRNA expression level of angiogenin-4 by qPCR. We found that angiogenin-4 expression was more than 100 fold higher in rIL-25 treated mice than in PBS treated mice (Fig 1A). We confirmed this pattern of expression by performing immunohistochemistry for angiogenin-4 in cecal tissue. Angiogenin-4 protein expression was highly upregulated in rIL-25 treated mice with expression observed in intestinal epithelial cells in the crypts and villi (Fig 1B–1D). We then tested for a dose dependent induction of angiogenin-4 by rIL-25. We found that 4 vs 8 doses of rIL-25 induced a 7-fold and 218-fold increase in angiogenin-4 compared to the PBS control (Fig 2). We concluded that IL-25 induced angiogenin-4 expression in the cecal intestinal epithelium.

### rIL-25 induces angiogenin-4 in an IL-13 dependent manner

**Fig 3:**
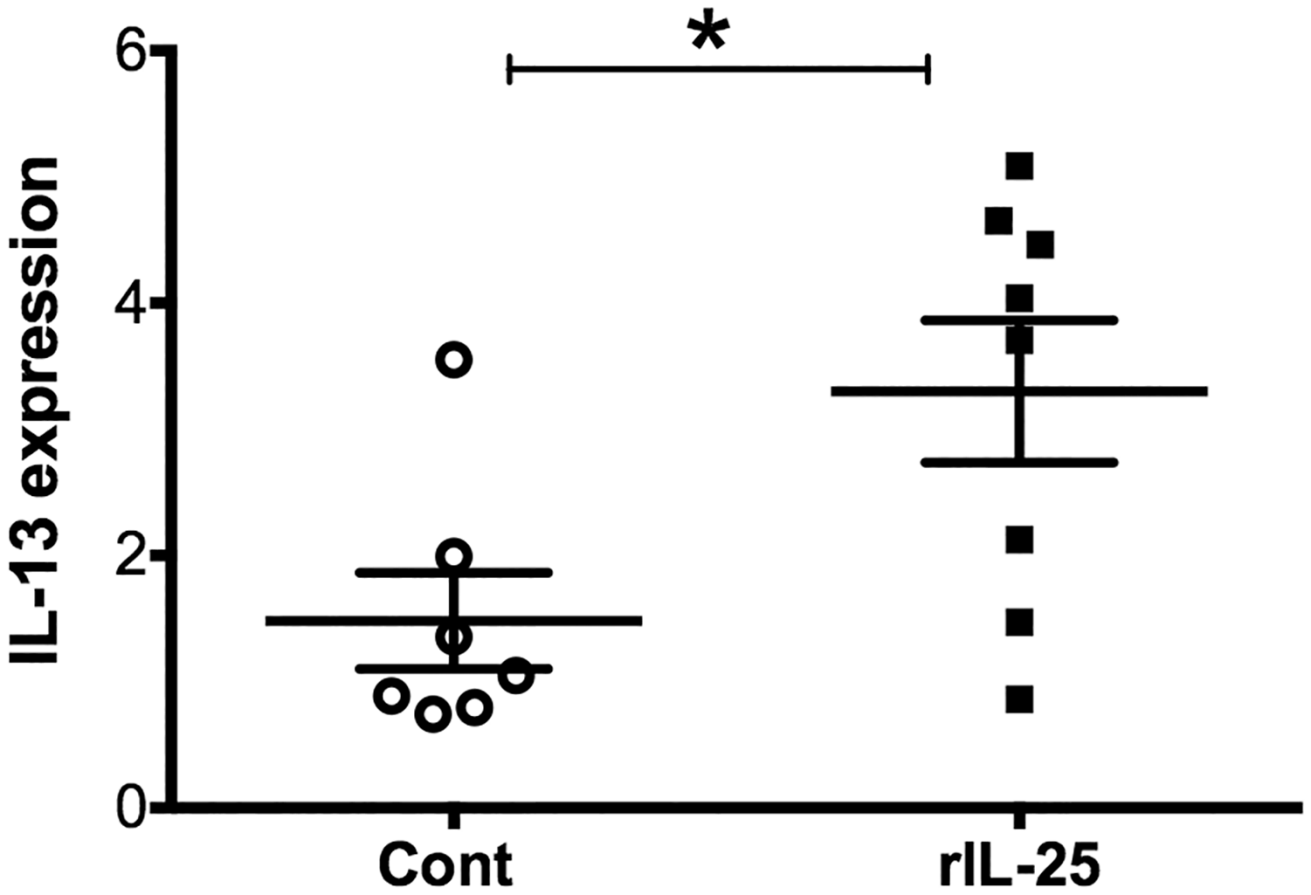
rIL-25 administration induces IL-13 expression. CBA/J mice were treated with 0.5 micrograms of recombinant IL-25 (closed square, n = 8) each day for a total of 5 doses and control mice received PBS (open circle, n = 7). IL-13 relative expression was measured from mouse cecal tissue and normalized with house keeping gene GAPDH and β actin.

**Fig 4:**
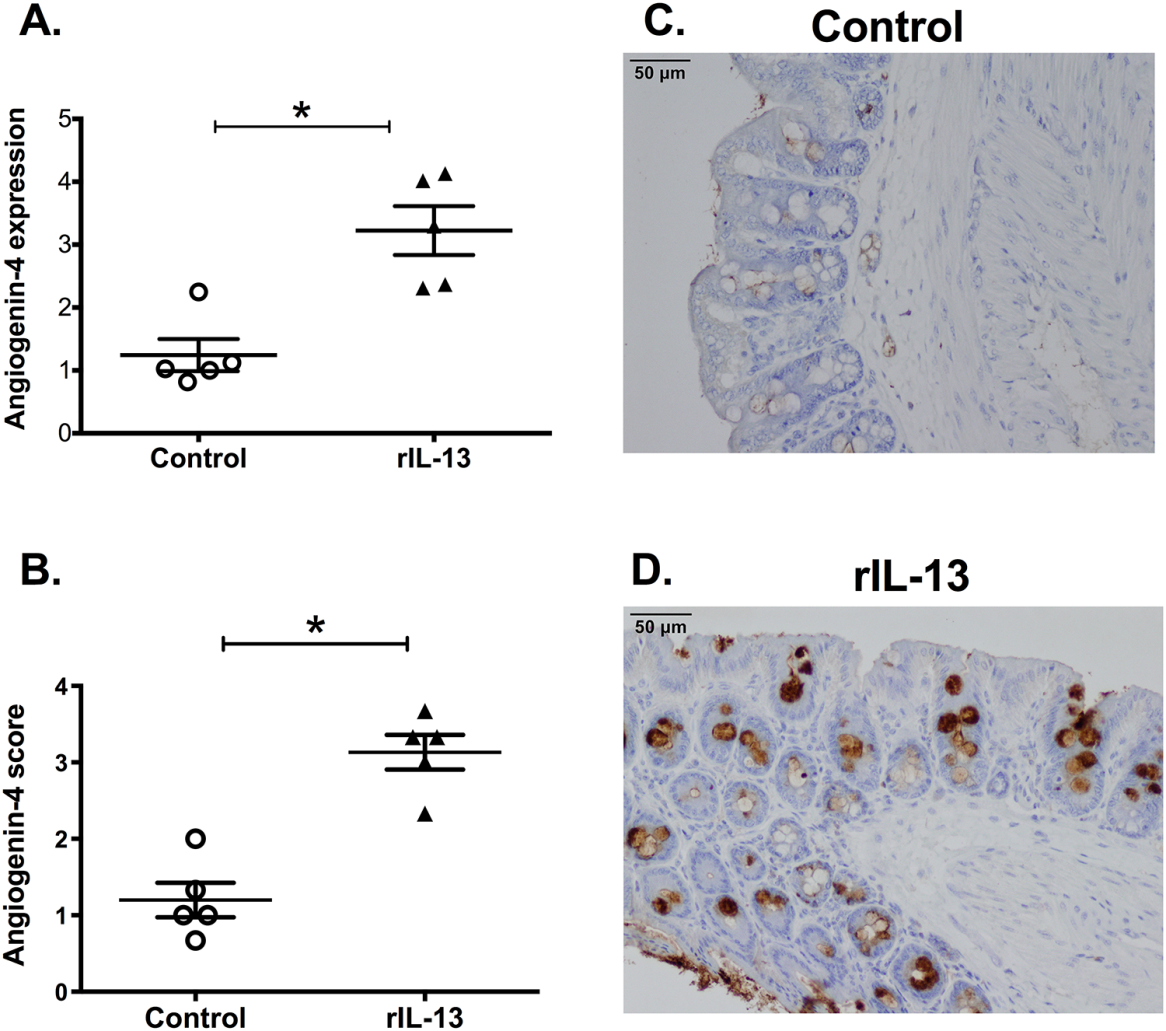
rIL-13 administration induces angiogenin-4 expression. CBA/J mice were treated with 0.5 micrograms of recombinant IL-13 (triangle, n = 5) on each day for total 4 doses. Control mice received PBS (open circle, n = 5). Angiogenin-4 relative expression was measured from mouse cecal tissue and normalized with house keeping gene GAPDH and β actin (A). Histological scoring (1 to 5; low to high) for Angiogenin-4 in mouse cecum from PBS or rIL-13 treated mice (B). Representative IHC staining for angiogenin-4 in samples of cecum tissue from PBS treated (C) or rIL-13 treated mice (D).

**Fig 5:**
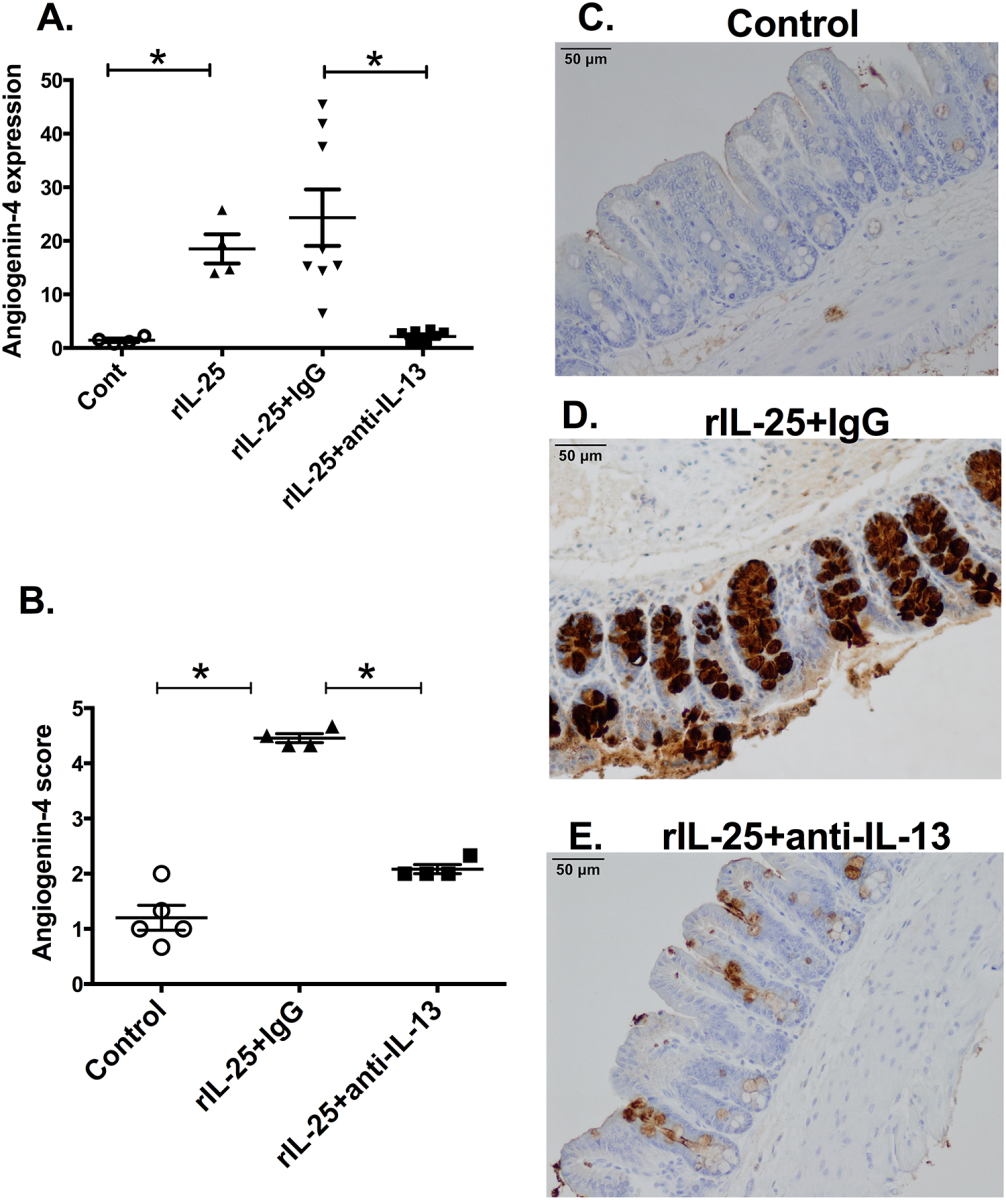
Depletion of IL-13 abrogates rIL-25 induction of angiogenin-4. CBA/J mice were treated with 0.5 micrograms of recombinant IL-25 each day for a total 6 doses. Control mice received PBS. Recombinant IL-25 treated mice received 200μg anti-IL-13 antibody or isotype control on day 3 and on day 5. Angiogenin-4 relative expression was measured from cecal tissue, n = 5–8 (A). Histological scoring (1 to 5; low to high) for Angiogenin-4 in cecum from mice treated with PBS or rIL-25 with isotype control or rIL-25 with anti-IL-13 (B). Representative IHC staining for angiogenin-4 in samples of cecum tissue from PBS treated (C) or rIL-25 with isotype control (D) or rIL-25 with anti-IL-13 treated mice (E).

IL-25 is known to induce Th2 cytokines, including IL-13. Therefore, the role of IL-13 in the ability of IL-25 to induce angiogenin-4 was examined. CBA/J mice were treated with rIL-25 (closed square, n = 8) and control mice received PBS (closed circle, n = 7). IL-13 relative expression was measured from mouse cecal tissue. We found that there was significantly higher expression of IL-13 in rIL-25 treated mice than that in control mice (Fig 3). Previous studies have shown that IL-13 can induce Paneth cell degranulation and trigger the release of the antimicrobial peptide angiogenin-4. We confirmed that IL-13 induced angiogenin-4 expression (Fig 4A) in cecal epithelial cells (Fig 4B–4D). IL-25 mediated angiogenin-4 expression was abrogated when IL-13 was depleted by neutralizing antibodies (Fig 5A). This result was confirmed via immunohistochemistry of angiogenin-4 as before (Fig 5B–5E). These results indicated that IL-25 induced angiogenin-4 expression was mediated via the IL-25 downstream pathway cytokine IL-13.

### IL-17 or IL-22 does not play a major role in IL-25 mediated angiogenin-4 induction

**Fig 6:**
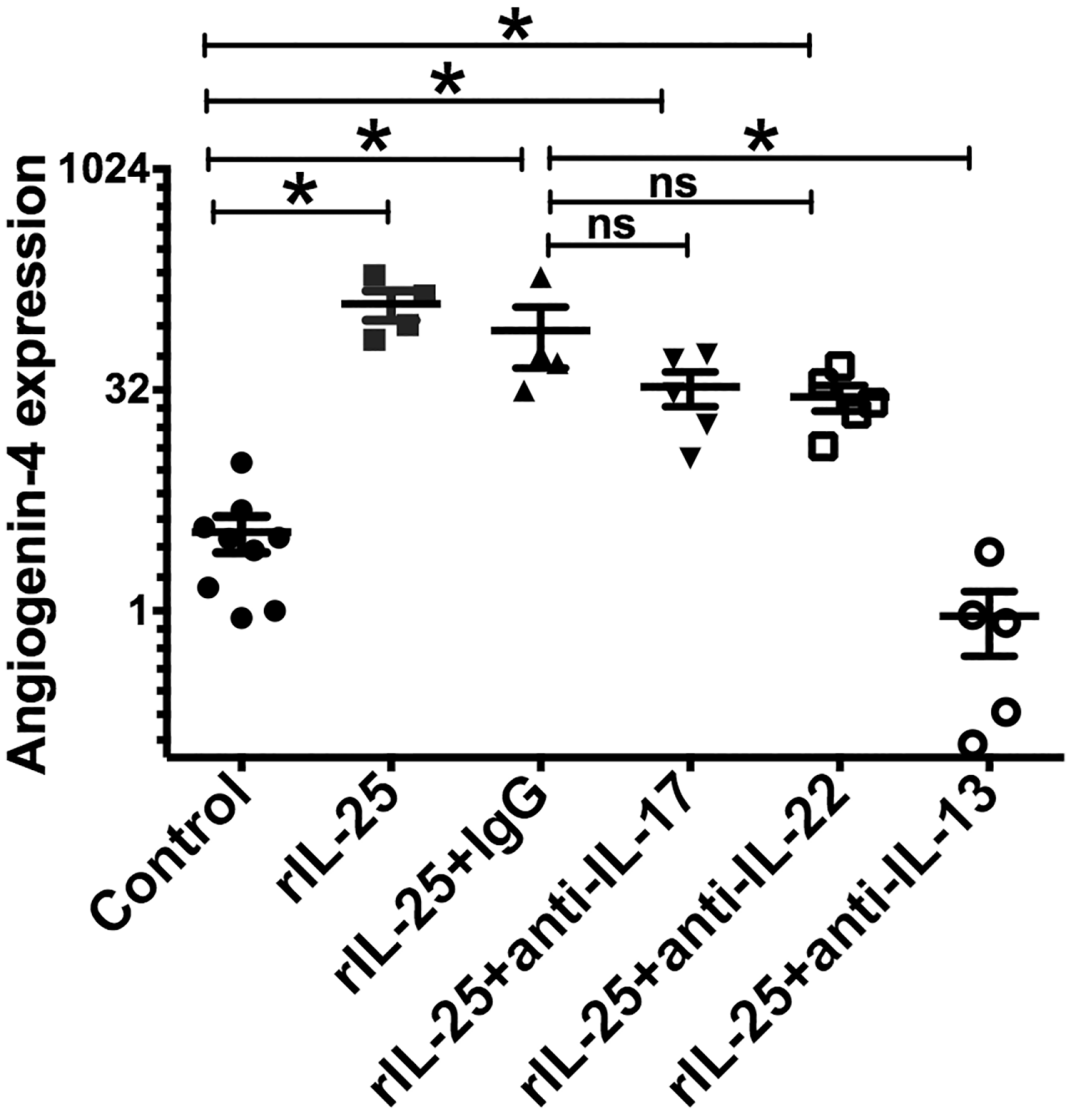
rIL-25 induced angiogenin-4 production is not significantly influenced by IL-17 or IL-22 blockade. CBA/J mice were treated with 0.5 micrograms of recombinant IL-25 each day for total 7 doses and control mice received PBS. rIL-25 treated mice received 200μg anti IL-17 antibody (inversed triangle) or 200μg anti IL-22 antibody (open square) or 200μg anti-IL-13 antibody (open circle), or isotype control on day 3, on day 5 and on day 7. Angiogenin-4 relative expression was measured from cecal tissue.

IL-17 and IL-22 are both known to be potent inducers of angiogenin-4 from Paneth cells during infection. We therefore tested the requirement of IL-22 or IL-17 in IL-25 mediated angiogenin-4 production. We depleted IL-22 or IL-17 by monoclonal antibodies in rIL-25 treated mice. We found that angiogenin-4 expression was partially decreased by the depletion of either IL-22 or IL-17 in rIL-25 treated mice, whereas IL-13 neutralization completely abrogated IL-25 induced angiogenin-4 expression (Fig 6). From these results, we concluded IL-25 induced angiogenin-4 expression largely depends on downstream IL-13, rather than IL-22 or IL-17.

## Discussion

IL-25 is known to have a protective role against helminth infections through the induction of a Th2 response. However its role in antimicrobial peptide induction, which can also play a role in gut barrier protection, has not been well studied. In the present study, we found that both IL-25 and IL-13 induce angiogenin-4 expression in the intestinal epithelium. Antibody-mediated neutralization of IL-13 blocked IL-25-mediated angiogenin-4 induction, demonstrating that IL-13 was acting downstream of IL-25.

Earlier results suggested that IL-13 is a key mediator of angiogein-4 production: both IL-13 and IL-4 trigger degranulation of antimicrobial peptides from Paneth cells [11]. The antimicrobial peptide angiogenin-4 is known to be induced by IL-9 in an IL-13 dependent way. It has also been shown that IL-13 knock out mice with suppressed angiogenin 4 failed to expel *T*. *muris* [5]. Other cytokines related to IL-25 that are known to induce antimicrobial peptides, include IL-22 and IL-17A [7, 12]. Therefore, we looked to see if angiogenin-4 expression by IL-25 is controlled by IL-22 or IL-17. When we measured angiogenin-4 expression in IL-25 treated and IL-22 or IL-17 neutralized mice, we found that IL-25 induced angiogenin-4 expression was not decreased.

Morphological analysis using immunohistological staining anti-agiogenin-4 could not reveal which cell type is responsible for IL-25 mediated angiogenin-4 expression. It would be interesting to know which cells are producing angiogenin-4 downstream of IL-25. IL-13 has been shown to play important roles in triggering degranulation of antimicrobial peptides from both Paneth cells and goblet cells [5,11], and both cells are present in the cecum. Therefore, it could be that IL-25 induces angiogenin-4 production from both cell types, and that IL-13 is needed for this. Future studies may examine this possibility.

In conclusion, our work makes the novel observation that IL-25 induces angiogenin-4 expression in a dose-dependent manner, and that IL-13 downstream of IL-25 is important in production of this antimicrobial peptide. This work better explains the sequence of events that underlies induction of angiogenin-4 by IL-25. The understanding of IL-25 regulation of the antimicrobial peptide angiogenin-4 may contribute to the understanding of its role in intestinal barrier protection, and in the development of therapeutic applications of IL-25 in the treatment of enteric diseases.

## Supporting Information

S1 Data Data tables supporting graphical figures. (XLSX)

S1 Fig Additional histology image set 1. (TIFF)

S2 Fig Additional histology image set 2. (TIFF)

S3 Fig Additional histology image set 3. (TIFF)

S4 Fig Additional histology image set 4. (TIFF)
